# 

**Published:** 1867-03

**Authors:** 


### CONTENTS.

ORIGINAL COMMUNICATIONS.

Address to the Graduating Class of the Ohio Dental College........ 89
Response of the Class........ 105

PROCEEDINGS OF SOCIETIES.

The Third Regular Semi-Annual Meeting of the Illinois State Dental Society........ 111

EDITORIAL.

Examinations for the Degree of D.D.S., in the Ohio College of Dental Surgery........ 137
Close of the College Session........ 142
Selfish........ 142
Dr. Brown's Dental Depot........ 143
Baltimore College of Dental Surgery........ 143
In Memoriam—A. M. Leslie, D. D. S........ 143

